# Effect of Grafting Conditions on the Interfacial Properties of Silane Modified Wood Veneer/PE Film Plywood

**DOI:** 10.3390/polym15132957

**Published:** 2023-07-05

**Authors:** Liang Chang, Yuanwu Wang, Xueqi Chen, Yiping Ren, Xiaoxi Luo

**Affiliations:** Research Institute of Wood Industry, Chinese Academy of Forestry, Beijing 100091, China; wangwu86295033@163.com (Y.W.); chenxueqi1995@163.com (X.C.); renyp600@163.com (Y.R.); 15600216715@163.com (X.L.)

**Keywords:** compatibilization, wood veneer, PE film, grafting conditions

## Abstract

In order to elucidate the importance of grafting in the compatibilization process of silane coupling agents, poplar veneer was treated with silane coupling agents and grafted under different heating conditions. The treated veneers were used composited with PE film to prepare different plywood samples. XPS and WCA were used to analyze the effect of grafting conditions on the surface properties of the silane-treated veneer. The results showed that free silanols can physically be adsorbed onto all silane-treated veneer surfaces, forming hydrogen–Si–O–Si– bonds and therefore increasing the water contact angle. Only under heating conditions could the –Si–O–Si– be converted into covalent –Si–O–C– bonds, which helped to improve the bonding strength. When silane-treated veneer was grafted at 120 °C for 90 min, the tensile shear strength of plywood reached 1.03 MPa, meeting the requirements of GB/T 9846.3-2004 for outdoor materials. Enhanced interlock between silane-modified veneer and PE film was observed under the optimal grafting condition by SEM. The better interface structure allowed improvement of thermal stability. DMA results showed that the retention rate in storage modulus at 130 °C was 60% for the grafted sample, while the retention rate for the ungrafted sample was only 31%.

## 1. Introduction

Plywood is widely used in furniture manufacturing, construction, and other fields due to its good physical and mechanical properties [1]. However, the adhesives used in the production of plywood are mainly urea formaldehyde resin (UF), phenolic resin (PF), and melamine formaldehyde resin (MF), which have different levels of formaldehyde (CH_2_O) release in the process of storage and use [2,3]. Great efforts have been made to reduce CH_2_O emission in aldehyde adhesives [4,5]. However, these methods, such as reducing the F/U mole ratio, cannot fundamentally solve the problem. Alternative “no-added-formaldehyde” adhesives, such as isocyanates (MDI), soy protein adhesives, and starch adhesives have also captured much attention [6,7,8]. However, their application was found to be limited due to their poor water resistance and high cost. In recent years, thermoplastic resin has attracted great attention because of its good flexibility, water resistance, and processability [9,10,11]. Thermoplastics are solvent-free and have various forms, such as granules, powders, sheets, films, wires, and blocks, which have the merit of unique adhesion performance when composited with wood components [12,13,14]. All forms of wood, such as wood veneer, wood particle, or wood fiber, can be directly bonded with thermoplastics. In most of the existing studies, thermoplastic films were preferentially selected to bond wood veneers to prepare wood–plastic plywood [15,16,17,18,19]. Thermoplastic films played a role of wood adhesives in the manufacturing process of plywood. Since the traditional formaldehyde adhesive has been completely replaced, the most remarkable feature of wood–plastic plywood is environmental friendliness. The results showed that the total volatile organic compound (TVOC) release rate and formaldehyde of wood–plastic plywood were 0.01 mg/(m^2^·h) and 0.2 mg/L respectively, which were far lower than the values specified in the relevant standards [20,21].

The study of thermoplastic as wood adhesive began in the 1990s and was first proposed by Dr. Han of Kyoto University [22]. Both virgin and recycled thermoplastics can be used to bond wood, but the melting temperature of the selected thermoplastic cannot be higher than 200 °C to avoid wood degradation. Polypropylene (PP), polyethylene (PE), polyvinyl chloride (PVC), polystyrene (PS), poly(lactic acid) (PLA), ethylene-vinyl acetate (EVA), and poly β-hydroxybutyrate (PHB) have been proven to be suitable alternative materials [15,18,19,23,24,25,26]. The manufacturing process of wood–plastic plywood is very simple. It only needs to be assembled with a thermoplastic film between every two wood veneers, and then fabricated using a combined hot-press and cold-press. In the preparation of wood–plastic plywood, the pressing conditions, film types, and thickness, as well as the wood surface properties directly affected the performance of plywood [27,28,29,30]. In general, the hot-pressing temperature should be 20–30 °C higher than the melting temperature of the thermoplastic film, so that it can fully flow into the porous structure of the wood. Luedtke et al. [24] found that the tensile strength of PLA plywood prepared at 160 °C was higher than that of plywood prepared at 140 °C. Physical–mechanical properties of wood–plastic plywood can meet the strength requirements for different applications by controlling the process conditions. Compared with natural-based adhesives, thermoplastic film can offer improved water resistance and relatively simple processing technology. However, the incompatibility between thermoplastic film and wood was not found to be conducive to the transfer of residual stress at the interface, thus limiting its application area.

A number of modification methods have been developed to improve the interfacial adhesion between wood and different thermoplastics. Physical treatments (e.g., thermal treatment) were shown to be relatively simple [31], but the enhancement effect was only average. Li et al. [26] punched holes on the surface of PVC film and then composited it with wood veneer. This method helped to accelerate the heat transfer efficiency and increase mechanical interlocking, but the self-strength of the wood veneer or plastic films was prone to deterioration. Endowing wood with new surface properties by chemical modification has been the most common and effective technique. Chemical modification, such as alkaline treatment [32], acetylation treatment [33], or by using coupling agents [34], can permanently alter the nature of the wood cell walls. Chemical modification is beneficial for improving the tensile strength of composites, but may sacrifice their impact strength. A coupling agent is a chemical that functions at the interface to create a chemical bridge between the wood and the thermoplastic. Extensively used coupling agents for wood–plastic composites have been maleated polypropylene (MAPP) [35], maleated polyethylene (MAPE) [36], isocyanate [37], or silane [38]. MAPP and MAPE have been commonly used to reinforce wood fibers and thermoplastics, as they require only mixing with raw materials to melt. The price of isocyanates is relatively high.

The bifunctional structures of silanes are particularly suitable for wood veneer modification because they can be configured as an aqueous solution. There are various types of silanes, such as aminosilanes, vinyl- and acryl-silanes, methacrylate-functional silanes, etc. Different thermoplastic resins need to be matched with different silane structures to achieve the reinforcement effects. In the existing literature, vinyl-silanes are the most extensively reported coupling agents between wood and PE. Our previous work showed the increased compatibility between wood veneer and PE film by spraying vinyltrimethoxysilane onto veneers [30]. Therefore, vinyltrimethoxysilane was selected in this work. Generally, the silane undergoes a hydrolysis process and generates reactive silanol groups at first. The reactive silanol monomers are then physically absorbed onto the hydroxyl groups of the wood by hydrogen bonding. Some of the free silanol groups also react with each other (self-condensation) at the same time. The hydrolysis process of silane has been comprehensively studied [39,40]. The competition of hydrolysis and silanol condensation can be controlled by the hydrolysis conditions. In order to minimize the condensation, hydrolysis in an acidic pH environment is usually recommended. Two competing reactions at different pH values were ascertained in situ using ^1^H, ^13^C, and ^29^Si NMR spectroscopy [41]. In addition, the grafting reaction initiated by heating after the deposition of silane on the veneer is also an essential step. During the grafting process, –Si–O–C–is formed. Although the –Si–O–C– linkages formed under heating condition were eventually not stable towards hydrolysis, researchers believe that it facilitated the enhancement of the interfacial adhesion between treated wood and thermoplastic, as well as improving the properties of the resulting composites. However, the effect of grafting conditions on the bridging effect of silane coupling agents is currently unclear. In this study, poplar veneer was sprayed with vinyltrimethoxysilane solution. The effects of heating temperature and time on the surface properties of silane modified poplar veneer and the mechanical properties of silane modified poplar/polyethylene plywood were analyzed.

## 2. Materials and Methods

### 2.1. Materials

Poplar veneers were purchased from Jicheng Decorative Materail Industry Co., Ltd. (Linyi, China) with a dimension of 400 × 400 × 1.7 mm. Their moisture content was controlled around 8%. Polyethylene film with a thickness of 0.05 mm was provided by Huadun Snowflake Plastic Group Co., Ltd. (Beijing, China). Vinyltrimethoxysilane was provided by Nanjing Chuangshi Chemical Additives Co., Ltd. (Nanjing, China) Ethanol (95%) and glacial acetic acid were purchased from Nanjing Jianghua Chemical Glass Co., Ltd. (Nanjing, China).

### 2.2. Surface Treatment

The pre-treatment process of poplar veneer was as follows: (1) Adjust the pH of 95% ethanol solution to pH 4 by adding glacial acetic acid; (2) Silane is then hydrolyzed in ethanol solution for 2 h to prepare the silane solution with a concentration of 4%; (3) Immerse the poplar veneer in the silane solution for 60 s and then dry at room temperature for 48 h; (4) The treated veneers were then divided into two groups and heated under different conditions to stimulate the grafting reaction. The first group was heated at 60, 80, 100, and 120 °C for 2 h respectively. The second group was heated at 120 °C, and the heating time was 30, 60, 90, and 120 min, respectively. At the same time, the unheated silane-treated veneer was set as the control sample (referred to as ungrafted).

### 2.3. Manufacturing of Wood Veneer/PE Film Plywood

**Figure 1:**
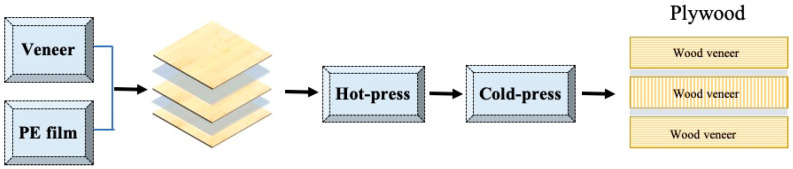
Manufacturing process of wood veneer/PE film plywood.

Three-layer plywood was assembled with one piece of PE film between every two poplar veneers (Figure 1). The plywood was first prepared with the grain directions of the two adjacent veneers perpendicular to each other. Then it was manufactured using a combination of hot-press and cold-press methods. The hot-press pressure, hot-press temperature, and hot-press time were 1 MPa, 155 °C, and 6 min respectively. In order to reduce the shrinkage and deformation of the PE film, the plywood was subjected to a secondary pressing at room temperature for 5 min, with the pressure controlled at 1 MPa.

### 2.4. Testing and Characterization

#### 2.4.1. X-ray Photoelectron Spectroscopy (XPS)

The surface chemical components of silane-treated poplar veneers under different grafting conditions were tested using XPS (Thermo ESCALAB 250 Xi spectrometer, Waltham, MA, USA). All spectra were collected using a monochromatic Al Kα X-ray source (hv = 1486.7 eV).

#### 2.4.2. Water Contact Angle (WCA)

Untreated wood veneer and silane-treated veneers under different grafting conditions were cut into 50 mm lengths and 10 mm in width. The image and WCA value were captured and recorded after the water drop (about 2 µL) was applied for 2 s using DSA-X ROLL (BeiTuo Science, Guangzhou, China). Each WCA measurement was made 3 times on each of the 5 wood specimens.

#### 2.4.3. Tensile Shear Strength

Tensile shear strength of the wood veneer/PE film plywood was evaluated using a multi-function mechanical testing machine (Jinan Shijin Co., Ltd., Jinan, China, 10 kN), with a crosshead speed of 10 mm·min^−1^ according to the Chinese National Standard (GB/T 17657-2013). Before the test was conducted, all specimens were first immersed in boiling water for 4 h, then dried at 63 °C for 20 h, and finally soaked in boiling water for 4 h with 20 observations per sample type.

#### 2.4.4. Scanning Electron Microscopy (SEM)

The plywood before the tensile test was cut into dimensions of 10 mm ×10 mm. The cross-section of the specimen was coated with gold and its interface morphology was examined using a Quanta-200 scanning electron microscope (Hillsboro, OR, USA).

#### 2.4.5. Dynamic Mechanical Analysis (DMA)

The DMA approach was applied (Q800, TA Instruments, New Castle, DE, USA) to test the storage modulus (*E*′) of different plywoods. The double cantilever bending mode with a span of 35 mm was selected. The temperature ramp experiment was conducted at a heating rate of 3 °C min^−1^ ranging from 30 to 200 °C, with an amplitude of 0.03 mm and a frequency of 1 Hz.

## 3. Results

### 3.1. X-ray Photoelectron Spectroscopy (XPS)

**Figure 2:**
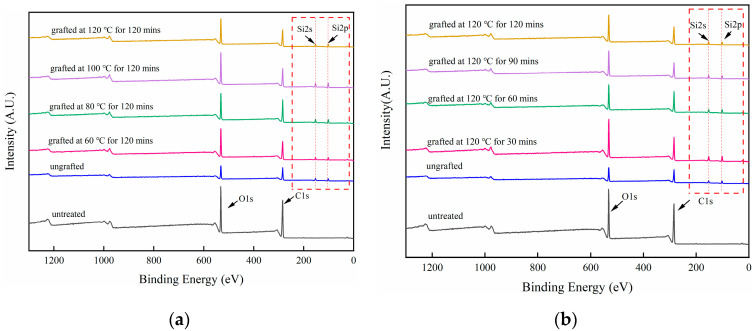
XPS spectra of silane-treated wood veneers under different grafting conditions: (**a**) different heating temperature, (**b**) different heating time.

**Figure 3:**
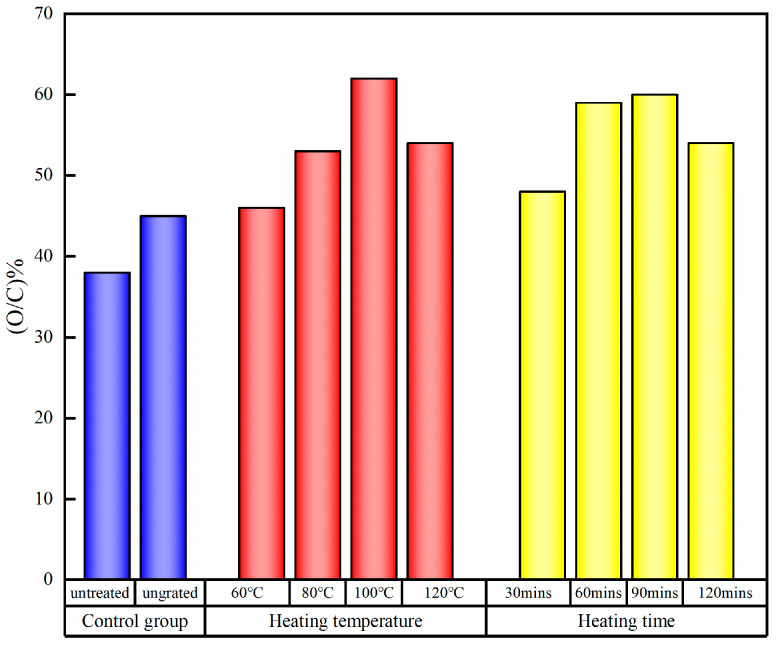
O/C ratio of silane-treated wood veneers under different grafting conditions.

XPS can not only be used to analyze the composition of chemical elements but to evaluate the chemical environment around the element according to the peak intensity and chemical shift of each element. As shown in Figure 2, two peaks at about 533 eV and 285 eV can be detected on the surface of the all the veneers by XPS. They represent oxygen (O) and carbon (C) atoms respectively [42,43]. Double characteristic emission peaks of silicon atoms were detected on all the silane-treated wood veneers, confirming the silane alcohol groups obtained through alkoxy hydrolysis can react with the hydroxyl groups of the wood surface or react by condensation onto the surfaces of the wood. After the hydrolyzed silane solution was deposited on the wood veneer, the O/C ratio increased due to the formation of –Si–O–Si– bonds. The grafting conditions affected the amount of silane adsorbed on the surface of the veneer after the plateau corresponding to the formation of a monolayer was reached. Temperature accelerated the self-condensation between the OH groups borne by the hydrolyzed silane molecules, and there was an interaction between temperature and time. As shown in Figure 3, when silane-treated veneer was heated at 100 °C for 2 h, the O/C ratio increased to 0.62, whereas the ratio slightly decreased when the heating temperature increased to 120 °C. This may be caused by the degradation of hemicellulose. At a heating temperature of 120 °C, the surface O/C ratio increased with the heating time.

**Figure 4:**
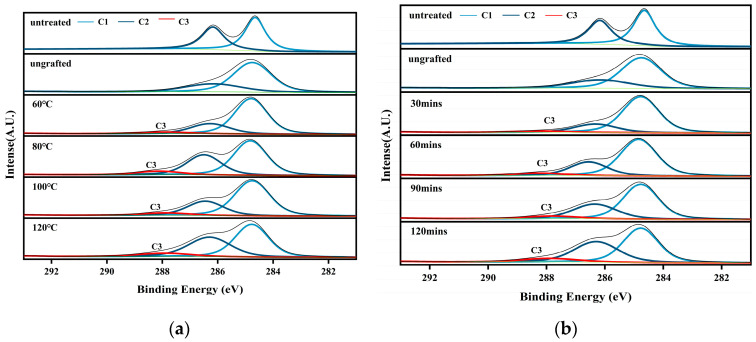
C spectra of poplar silane-treated veneer under different grafting conditions: (**a**) different heating temperature, (**b**) different heating time.

**Figure 5:**
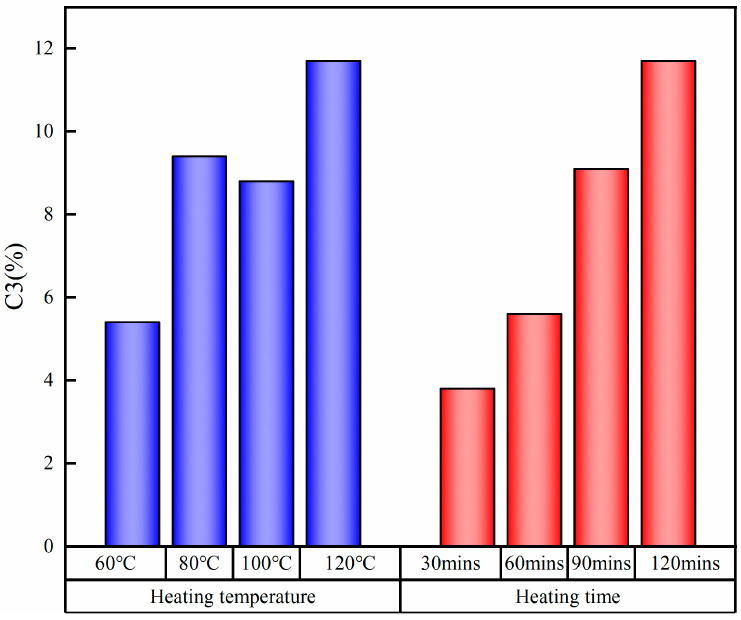
C3 content of silane-treated wood veneers under different grafting conditions.

The detailed features of C1s for different wood veneers are shown in Figure 4. There were two main structures on the surface of wood: C1 and C2. C1 came from extracts of terpenoids and fatty acids in wood, as well as phenylpropane in lignin, corresponding to the C–H or C–C bond. C2 corresponded to the C–O bond, and the carbon atom connecting with hydroxyl in the cellulose and hemicellulose in the veneer belonged to this type of structure. When the silane-treated veneer was heated at a certain temperature, a new C3 structure (–Si–O–C–) formed. This was consistent with previous research [43,44]. As shown in Figure 5, the proportion of C3 increased with the heating temperature. When the grafting condition was fixed at 120 °C and 2 h, the content of C3 was the highest. Shortening the heating time reduced the content of C3 when the heating temperature was fixed at 120 °C. It should be noted that a decreasing trend was observed in the C3 proportion at 100 °C. This was because the dehydration and self-condensation reactions competed with each other during the heating process. The self-condensation reaction may dominate at 100 °C. However, this C3 structure was not observed in the ungrafted veneer. The main reason for this phenomenon is that the hydrogen bonds formed between the adsorbed silanols and the hydroxyl groups of the wood veneers at the adsorption sites cannot convert into covalent bonds at room temperature [45].

### 3.2. Water Contact Angle (WCA)

**Figure 6:**
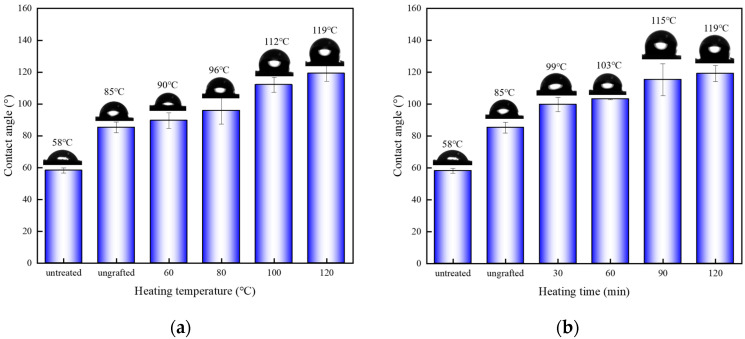
WCA value of silane-treated veneers under different grafting conditions: (**a**) different heating temperature, (**b**) different heating time.

Figure 6 shows the effect of grafting conditions on the wettability of poplar veneer. The WCA value of unmodified veneer was about 58°. Due to the formation of polysiloxane multilayers on the veneer surface, the normally hygroscopic wood was gradually converted into a hydrophobic form after silane modification. As shown in Figure 6a, the heating temperature had a positive effect on the WCA value of the wood veneer. When the heating temperature increased from 60 °C to 120 °C, the WCA value increased from 90° to 119°, and the shape of water droplets gradually changed from semicircular to elliptical. This was attributed to the hydrophobic silane coating deposited on the surface of the veneer, while another important reason was that the veneer underwent a process similar to heat treatment during grafting at the higher temperature conditions. When wood veneer was heated at high temperature, the free –OH between the cellulose molecular chains underwent a “bridging” reaction, forming hydrogen bonding and reducing the number of –OH groups. The longer the heating time, the bigger was the WCA value. The WCA value gradually decreased with the reduction of heating time when the heating temperature was fixed at 120 °C. As shown in Figure 6b, the value was 99° when the heating time was kept at 30 min, which was only 16% higher than the ungrafted sample.

### 3.3. Tensile Shear Strength

**Figure 7:**
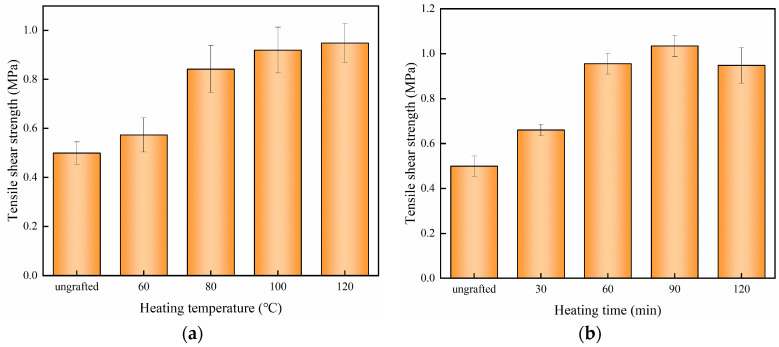
Tensile shear strength of silane-modified plywood under different grafting conditions: (**a**) different heating temperature, (**b**) different heating time.

**Figure 8:**
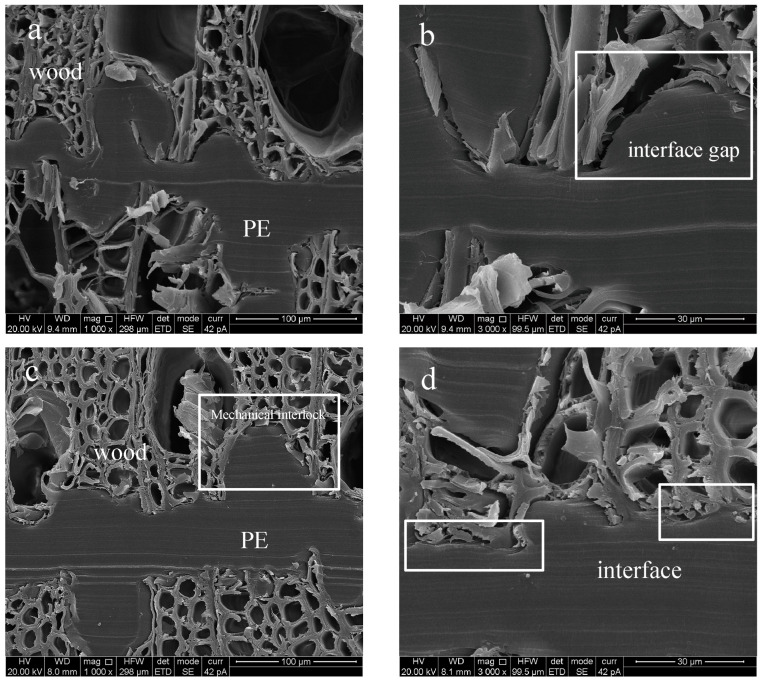
SEM images of interface between polyethylene and silane-treated veneer: (**a**) ungrafted ×1000, (**b**) ungrafted ×2000, (**c**) grafted at optimum conditions ×1000, (**d**) grafted at optimum conditions ×3000.

In the manufacturing process of plywood, the PE film melts and infiltrates into the porous structure of the wood veneer, then cools and solidifies to form a “glue nail” microstructure. The microstructure is stable and can give plywood a certain strength [15], but the adhesive layer of unmodified plywood completely peels off after soaking in boiling water. The poor boiling water resistance is mainly caused by the incompatibility between PE and wood veneer. In our previous studies, a significant gap at the interface was observed [30]. Silane treatment can enhance interfacial adhesion, and the grafting conditions greatly affects the modification effect. As shown in Figure 7, if the silane-modified veneer was not heated or heated at a lower temperature before being composited with the PE film; the tensile shear strength was lower than 0.7 MPa, which cannot meet the requirements of GB/T 9846.3-2004 for outdoor plywood. Figure 8a,b shows the SEM of ungrafted plywood. Although the adhesive layer is continuous and uniform, defects in the bonding structure can still be observed at high magnification.

When the heating temperature increased to 120 °C, the tensile shear strength reached 0.95 MPa. This resulted from the –Si–O–C– bonds formed under heating condition, which facilitated enhancement of the interfacial adhesion between the silane-treated veneer and the PE film, as well as the bonding strength of the resulting plywood. The higher the heating temperature, the more fully was the covalent bridging effect of –Si–O–C–taking place. The result is consistent with the previous analysis of XPS and WCA. The role of –Si–O–C– bonds can be more intuitively seen in Figure 8c,d. When the silane-treated wood veneer is heated at 120 °C, it can be tightly bonded with PE, resulting in a much more stable bonding structure. No gaps were observed even at a magnification of 3000 times. The tensile shear strength of plywood showed an upward trend with the increase of heating time when maintaining the heating temperature at 120 °C. The highest strength was obtained when the heating time was 90 min, as shown in Figure 6b. This was because the longer the heating time, the more –Si–O–C– formed on the surface of the veneer, ensuring the best mechanical properties for the plywood. However, due to the loss of its own strength of wood veneer, there was a slight decrease in the strength when the heating time increased to 120 min.

### 3.4. DMA

**Figure 9:**
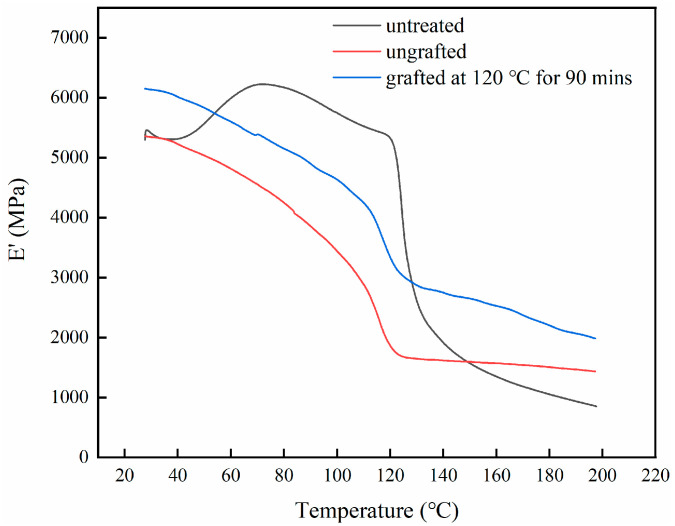
Storage modulus vs. temperature of different plywoods.

Since PE is a thermoplastic material, the adhesive layer formed between the PE film and the veneer is easily damaged at higher temperatures. This results in a loss of overall strength of the plywood. In order to investigate the effect of interface improvement on thermal stability, DMA was used to test the changes in storage modulus (*E*′) of different plywoods in the range of 30–200 °C, as shown in Figure 9.

The *E*′ of the three types of plywood decreased with the increase of temperature. As presented in Table 1, when the temperature was below 100 °C, the retention rate of untreated plywood *E*′ could be maintained at around 70%. This was because the PE had not yet begun to melt and soften within this temperature range. When the temperature reached 120 °C, the retention rate remained at only 20% due to sliding of the bonding interface. The strengthening effect of the silane coupling agent on the interface helped to improve the thermal stability of the plywood. The positive effect of silane treatment can be clearly observed when the temperature was above 120 °C. When silane-treated veneer was grafted at 120 °C for 90 min, the retention rate of the plywood at 130 °C increased to 60%. Consistent with the results of interface changes, if the silane modification was performed on the veneer without subsequent heating steps, the improvement effect on the thermal stability was weakened. The E′ retention rate of the ungrafted plywood at 130 °C was 31%. When the temperature reached 200 °C, the rigidity of all samples sharply decreased due to severe slip and damage at the bonding interface. Even under the optimal grafting conditions, the *E*′ retention rate of silane modified samples was less than half.

## 4. Conclusions

Pre-treatment of poplar veneer with vinyltrimethoxysilane is a simple way to improve the bonding strength and heat resistance of wood veneer/PE film plywood. The covalent bond of the –Si–O–C– linkage plays an important role in enhancing the interfacial adhesion between silane-treated wood and PE film. However, the hydrogen bonds formed between the adsorbed silanols and hydroxyl groups of wood veneers at the adsorption sites cannot be converted into –Si–O–C–bonds at room temperature. Therefore, the silane-modified veneer must undergo heating treatment in order to be composited with PE film. The generation rate of –Si–O–C– increases with the heating temperature and heating time. When the grafting conditions were controlled at 120 °C for 90 min, –Si–O–C– could be formed on the veneer surface without causing a decrease in the strength of the wood itself. Under optimal conditions, the plywood can meet the requirements for outdoor materials according to GB/T 9846.3-2004.

## Figures and Tables

**Table 1:** *E*′ retention rate of different plywood samples.

Sample Type	*E*′ Retention Rate at Different Temperatures (%)					
	60 °C	80 °C	100 °C	120 °C	130 °C	200 °C
untreated	89	78	67	23	19	13
ungrafted	90	79	64	35	31	27
grafted (120 °C, 90 min)	92	86	79	66	60	39

## Data Availability

Not applicable.
